# Isolation and characterization of microsatellite loci from *Oxytropis diversifolia* (Fabaceae)

**DOI:** 10.1002/aps3.1168

**Published:** 2018-07-11

**Authors:** Hui Wang, Han Yang, Pei‐Liang Liu, Chun Su, Liang Xiao, Zhao‐Yang Chang

**Affiliations:** ^1^ College of Life Sciences Northwest A&F University Yangling 712100 Shaanxi People's Republic of China; ^2^ Key Laboratory of Resource Biology and Biotechnology in Western China Northwest University Xi'an 710069 Shaanxi People's Republic of China

**Keywords:** Fabaceae, next‐generation sequencing, nuclear microsatellites, *Oxytropis diversifolia*

## Abstract

**Premise of the Study:**

Microsatellite primers were developed for a perennial legume from northern China, *Oxytropis diversifolia* (Fabaceae), to investigate population genetic structure of this taxon, as well as potential hybridization events with closely related taxa in this genus.

**Methods and Results:**

One hundred and five primer pairs were designed from Illumina sequence data and screened for suitability. Fifteen of these primer pairs were polymorphic, and these primers amplified tri‐, tetra‐, and pentanucleotide repeats with 10–56 alleles per locus. Cross‐amplification tests in three other *Oxytropis* species from northern China (*O. leptophylla*,* O. neimonggolica*, and *O. squammulosa*) revealed that all of these loci can be amplified successfully and show polymorphism.

**Conclusions:**

These primer pairs can be used to assess the genetic diversity and population structure in future studies of *O. diversifolia*, as well as studies of potential hybridization events with closely related taxa in this genus.

*Oxytropis diversifolia* E. Peter (Fabaceae) is a perennial herb occurring in dry *Stipa* L. grasslands/semi‐desert regions of northern China and Mongolia (Zhu et al., 2010). In the Nei Mongol region of China, populations of this species show morphological variation for leaf shape: individuals may have leaves with only one leaflet, three leaflets, or one to three leaflets. Because of the essential role that leaves play in photosynthesis, it is broadly accepted that variation in leaf shape has major ecological and evolutionary consequences, and such a character is expected to experience different selection depending on environmental conditions (Nicotra et al., 2011). However, direct evidence to demonstrate that leaf shape is actually adaptive is comparatively rare (Kidner and Umbreen, 2010). Intraspecific phenotypic variation in leaf shape could simply be the result of either random genetic drift or indirect selection on genetically correlated characters. A classical approach to assess the roles of purely neutral processes and natural selection in phenotypic differentiation is to compare the geographic pattern for the trait of interest to a set of putatively neutral loci (e.g., allozymes, microsatellites, amplified fragment length polymorphism [AFLP] markers, and single‐nucleotide polymorphism [SNP] markers). Currently, random‐amplified polymorphic DNA (RAPD) and AFLP markers have been developed and used in *Oxytropis* DC. species (e.g., Chung et al., 2004), but microsatellite markers are lacking.

Here, we describe the development of microsatellite markers that will facilitate future research on leaf shape variation in *O. diversifolia*. In addition, the degree of congeneric cross‐transferability of the markers was also assessed in three related *Oxytropis* species from northern China: *O. leptophylla* (Pall.) DC., *O. neimonggolica* C. W. Chang & Y. Z. Zhao, and *O. squammulosa* DC. We are particularly interested to test for potential hybridization of *O. leptophylla* with *O. diversifolia* (H. Wang, Northwest A&F University, Yangling, Shaanxi, China, personal observation).

## METHODS AND RESULTS

Total genomic DNA was extracted from a dry leaf sample collected in Urad Zhongqi, Nei Mongol, China (Pop8, Appendix 1; BioSample accession SAMN08408037), using the cetyltrimethylammonium bromide (CTAB) method (Doyle and Doyle, 1990). A DNA library was constructed with the KAPA Hyper Prep Kit (catalog no. KK8500; Kapa Biosystems, Wilmington, Massachusetts, USA), and 2 × 150‐bp paired‐end sequencing was performed on an Illumina HiSeq 2500 system (Illumina, San Diego, California, USA) at the Sequencing and Genotyping Facility of Beijing Microread Gene Technology Co. Ltd. (Beijing, China). A total of 3,421,900 raw sequence reads (2.52 Gbp, GenBank Short Read Archive accession SRP131738, BioProject ID PRJNA431827) were obtained. The paired‐end reads were then processed using Trimmomatic version 0.35 (Bolger et al., 2014) and merged into ~240‐bp sequences using FLASH version 1.2.11 (Magoč and Salzberg, 2011). In total, 3,079,710 clean reads assembled into 2,949,319 contigs.

SSR_pipeline software (Miller et al., 2013) was used to detect tri‐, tetra‐, and pentanucleotide repeats on the sequence set, and Primer Premier 5.0 (PREMIER Biosoft International, Palo Alto, California, USA) was used to develop primers for 105 loci, prioritizing motif diversity and melting temperature difference ≤1°C. An M13 tag (5′‐TGTAAAACGACGGCCAGT‐3′) was added to the 5′ end of the shorter primer of each locus. These primer pairs were tested on seven *O. diversifolia* individuals from different populations (Appendix 1). Each locus was initially amplified individually in 15‐μL PCR reactions that contained 1.5 μL of 10× Buffer I, 200 μM of dNTPs, 0.27 μM of M13‐tailed primer, 0.07 μM of untailed primer, 0.27 μM of M13 primer (labeled with HEX), 0.1 μL of 1× TaKaRa HS Taq (TaKaRa Biotechnology, Dalian, Liaoning, China), and 1.2 μL of diluted template DNA. PCR thermocycling conditions were an initial denaturation of 95°C for 5 min; 30 cycles of 94°C for 30 s, 56°C for 30 s, and 72°C for 30 s; followed by 10 cycles of 94°C for 30 s, 53°C for 30 s, and 72°C for 30 s; and a final extension at 60°C for 30 min. The PCR products were examined on a 2% agarose gel. Samples that yielded products of expected size were then submitted with a GeneScan 500 LIZ Size Standard (Applied Biosystems, Foster City, California, USA) for genotyping on an ABI 3730xl DNA Analyzer (Applied Biosystems). Resulting chromatograms were scored using GeneMapper version 3.2 (Applied Biosystems).

Of the 105 primer pairs tested, 15 produced repeatable amplicons and showed polymorphism across all seven individuals (Table 1). These 15 pairs were subsequently screened using four populations of *O. diversifolia* (*n* = 20, 30, 32, 32, respectively) and additional populations of *O. leptophylla* (*n* = 19), *O. neimonggolica* (*n* = 20), and *O. squammulosa* (*n* = 16) (Appendix 1). Instead of M13‐tailed primers, we used primers with a 5′ fluorophore, labeled with 6‐FAM, HEX, or ROX (Applied Biosystems, for specific fluorescent dye used for each locus see Table 1). Primer sequences and allele ranges for validated loci were fed into Multiplex Manager (Holleley and Geerts, 2009) to determine the best sets of loci available to include in a multiplex protocol (see Table 1 for pooling groups). The PCR reactions were performed separately for each locus, and the PCR products were then pooled into four groups for genotyping. The 15‐μL PCR reactions contained 7.5 μL of 2× TSINGKE Master Mix (Tsingke Biological technology, Xi'an, Shaanxi, China), 0.67 μM of forward primer, 0.67 μM of reverse primer, and 1 μL of diluted template DNA. The PCR thermocycling conditions and genotyping method were the same as above. Resulting chromatograms were scored using Geneious version 9.0.2 (http://www.geneious.com; Kearse et al., 2012).

**Table 1:** Characteristics of 15 microsatellite loci developed for *Oxytropis diversifolia*.^a^

Locus^b^	Primer sequences (5′–3′)	Repeat motif	Allele size range (bp)	Fluorescent dye (Group)	GenBank accession no.
N745892	F: CGGGTAGATTTCAGTTTTTGC	(ATAG)_12_	156–274	6‐FAM (1)	MG693777
	R: TGGGTCCCACTTATCACATTATC				
N145635	F: CCTGGGCTGAGAGAAGAAGA	(GAG)_12_	102–207	HEX (1)	MG693767
	R: TTCTCACGCTCATTTTGACG				
N2724893	F: ACTAATGGCCAGACCATATTCA	(AAC)_10_	108–138	ROX (1)	MG693774
	R: TACCTGACTTACCTTTGGGACA				
N876535	F: GAGGGAAGGGGAAAGTGAGA	(TCTA)_10_	126–322	ROX (1)	MG693779
	R: CGGATCCGGTTAACCTCTAA				
N2720763	F: CGCCGTTGATGAGTAACCTT	(TTCT)_10_	119–215	6‐FAM (2)	MG693773
	R: GAAAACAGATCGGGAATCCA				
N2717495	F: CCACAATCAAATTTTGGACG	(TCTA)_10_	144–198	HEX (2)	MG693772
	R: GGAGTGGTTGTTTTGATGAAAGT				
N178451	F: TCAGCATCATTTCCCAATCA	(ATATA)_13_	97–183	ROX (2)	MG693769
	R: GGGAATATAGAAAGTATCCACTGC				
N161850	F: CCGTGCATCAACCTAATGGT	(AAT)_13_	105–180	6‐FAM (3)	MG693768
	R: CCAACAACTTCTCCTTTGCG				
N49251	F: CCATGCAGCAGCTCTACAAA	(TCT)_11_	103–136	HEX (3)	MG693776
	R: GGAGTACGAAATCGGCGTTA				
N350553	F: TCAATTTCCATCTCGTGAACC	(TTC)_22_	130–280	HEX (3)	MG693775
	R: TGAGGTCATCACTCCATCAGA				
N935993	F: GATCATCGTGGTGATGATGG	(ATG)_10_	90–117	ROX (3)	MG693780
	R: CGCACTACCACCCTCTGAAT				
N1172223	F: TGGGATATGGAGGATGTCAG	(ATA)_15_	107–197	ROX (3)	MG693766
	R: GACCACCCCGTCAATCATAG				
N803014	F: CTGAGTAGAGAGTTCACAGGTCATGG	(AAT)_14_	125–197	6‐FAM (4)	MG693778
	R: TGATTTACCCATAGCAAGCAGA				
N2528349	F: TCTCTCTAATGGATTCCAGAACG	(ATCT)_20_	136–224	HEX (4)	MG693770
	R: TGGAGATGATGAAAGCACCA				
N2697375	F: TTGCCTTCAGTTTTGGGGTA	(TATG)_15_	141–238	ROX (4)	MG693771
	R: TCAAAGAGGGAAAACTGGGA				

a All values are based on 114 samples representing four populations (Baotoubei, Pop8, Hu, Dian1) located in dry grassland/semi‐desert regions of northern China. For details of voucher and locality information, see Appendix 1.

b Annealing temperature was 56°C for all loci.

Genetic diversity parameters were calculated using GenAlEx version 6.503 (Peakall and Smouse, 2006, 2012). Observed and expected heterozygosity levels ranged from 0.048 to 0.897 and 0.567 to 0.968, respectively (Table 2). Alleles per locus ranged from 10 to 56 in *O. diversifolia*. Tests of pairwise linkage disequilibrium were performed using GENEPOP 4.7 (Rousset, 2008). Only two genotypic disequilibria out of 420 (N876535 and N2720763, N876535 and N350553 in Pop8) were significant at the 5% level after Benjamini–Hochberg correction (Benjamini and Hochberg, 1995). We also used exact tests implemented by GENEPOP software to test for departure from Hardy–Weinberg equilibrium (HWE). A significant departure from HWE was recorded for almost all loci across the four populations (Table 2). Nine of the 15 loci failed to meet HWE expectations in at least one population. MICRO‐CHECKER (van Oosterhout et al., 2004) identified the possibility of null alleles in some loci. Three loci (N2724893, N350553, N803014) showed evidence of stuttering in some populations, but not consistently across populations, indicated as a deficit of heterozygote genotypes with alleles of one repeat unit difference. No large allele dropouts were identified. These departures from HWE are likely due to inbreeding or genetic drift.

**Table 2:** Genetic properties of the 15 polymorphic microsatellite loci in *Oxytropis diversifolia*.^a^

	Baotoubei (*n* = 20)			Pop8 (*n* = 30)			Hu (*n* = 32)			Dian1 (*n* = 32)			Total (*n* = 114)
Locus	*A*	*H*_o_	*H*_e_ b	*A*	*H*_o_	*H*_e_ b	*A*	*H*_o_	*H*_e_ b	*A*	*H*_o_	*H*_e_ b	*A*_T_
N745892	11	0.550	0.864***	17	0.600	0.889***	17	0.563	0.921***	21	0.710	0.937***	29
N145635	8	0.111	0.832***	16	0.517	0.877***	19	0.690	0.928***	18	0.645	0.906***	29
N2724893	6	0.750	0.728^ns^	7	0.433	0.699**	8	0.452	0.759***	8	0.406	0.584*	11
N876535	10	0.316	0.868***	27	0.724	0.948***	28	0.781	0.968***	28	0.531	0.960***	56
N2720763	8	0.421	0.852***	12	0.567	0.866***	10	0.438	0.880***	10	0.400	0.871***	13
N2717495	9	0.278	0.876***	14	0.241	0.859***	18	0.531	0.925***	18	0.367	0.936***	25
N178451	9	0.368	0.836***	14	0.800	0.873*	12	0.688	0.850*	12	0.563	0.900***	18
N161850	8	0.850	0.837^ns^	18	0.897	0.907^ns^	21	0.844	0.924**	16	0.839	0.901*	25
N49251	8	0.750	0.791^ns^	9	0.733	0.815^ns^	9	0.871	0.876^ns^	8	0.750	0.813^ns^	11
N350553	7	0.313	0.788***	21	0.500	0.930***	14	0.469	0.764***	16	0.467	0.823***	35
N935993	5	0.600	0.601^ns^	8	0.567	0.767*	10	0.781	0.785^ns^	8	0.581	0.717*	10
N1172223	12	0.700	0.885**	17	0.444	0.924***	19	0.533	0.925***	18	0.483	0.947***	26
N803014	3	0.412	0.597*	9	0.048	0.817***	10	0.240	0.857***	10	0.160	0.829***	16
N2528349	7	0.368	0.812***	14	0.600	0.788***	6	0.469	0.567^ns^	7	0.531	0.589*	18
N2697375	8	0.750	0.835^ns^	17	0.552	0.898***	18	0.677	0.921***	16	0.633	0.900***	31

Note

*A* = number of alleles detected across all individuals; *A*_T_ = total number of alleles; *H*_e_ = unbiased expected heterozygosity; *H*_o_ = observed heterozygosity; *n* = number of individuals sampled.

a Voucher and locality information are provided in Appendix 1.

b Statistically significant deviation from Hardy–Weinberg equilibrium is indicated as *P < 0.05, **P < 0.01, ***P < 0.001; ns = not statistically significant (*P* > 0.05).

Cross‐amplification of the 15 primer pairs was conducted on three related species from northern China: *O. leptophylla*,* O. neimonggolica*, and *O. squammulosa* (Appendix 1). All of the loci were successfully amplified and polymorphic. The number of alleles per locus varied from two to 22 in *O. leptophylla*, three to 21 in *O. neimonggolica*, and two to 18 in *O. squammulosa* (Table 3).

**Table 3:** Cross‐amplification of the 15 microsatellites developed for *Oxytropis diversifolia* in *O. neimonggolica*,* O. leptophylla*, and *O. squammulosa*.^a^

	*O. leptophylla* (*n* = 19)		*O. neimonggolica* (*n* = 20)		*O. squammulosa* (*n* = 16)	
Locus	*A*	Allele size range (bp)	*A*	Allele size range (bp)	*A*	Allele size range (bp)
N745892	13	188–250	17	154–262	10	160–234
N145635	6	114–129	18	99–228	4	99–117
N2724893	6	120–135	7	120–138	7	117–138
N876535	16	154–316	20	135–226	18	194–310
N2720763	5	183–215	9	141–207	2	167,171
N2717495	11	144–210	15	160–202	4	164–170
N178451	11	103–158	10	103–168	7	103–158
N161850	22	102–171	19	102–192	7	105–177
N49251	4	106–118	6	112–130	8	112–133
N350553	2	172, 178	21	136–262	6	172–187
N935993	3	102, 105, 108	6	96–111	3	99, 105, 111
N1172223	6	125–179	16	131–203	14	137–203
N803014	4	134–215	3	134, 137, 140	6	134–149
N2528349	10	150–244	6	149–184	3	154, 158, 162
N2697375	10	157–205	17	161–209	3	161, 167, 205

Note

*A* = number of alleles detected across all individuals; *n* = number of individuals sampled.

a Voucher and locality information are provided in Appendix 1.

## CONCLUSIONS

The 15 polymorphic microsatellites developed here will be used for population genetic studies on *O. diversifolia*. Cross‐amplification experiments confirmed that these markers should be applicable in *O. neimonggolica*,* O. leptophylla*, and *O. squammulosa*, thus providing a novel population genetic tool in *Oxytropis*. The low‐genomic‐coverage Illumina sequencing reads generated in the present study could potentially be used to assemble high‐copy‐number gene regions, such as complete or partial chloroplast and mitochondrial genomes, as well as nuclear ribosomal RNA genes. Such gene sequences can be informative in phylogenetic reconstruction of the genus *Oxytropis*, or even in a much broader phylogenetic scope.

## AUTHOR CONTRIBUTIONS

H.W. designed the experiment, collected samples in the field, analyzed the data, and wrote the manuscript. H.Y. did the molecular work under the supervision of H.W. P.‐L.L. was responsible for the field work, collected the voucher specimens, and participated in writing the manuscript. C.S. and L.X. participated in the field work. Z.‐Y.C. supervised the entire study. All authors approved the final version of the manuscript.

## DATA ACCESSIBILITY

Next‐generation sequencing data: (1) BioSample accession SAMN08408037, (2) BioProject ID PRJNA431827, (3) GenBank Short Read Archive accession SRP131738. Sequence data for the 15 microsatellite loci were submitted to GenBank, and accession numbers are listed in Table 1.

## APPENDIX 1 Voucher and locality information for *Oxytropis* species used in this study.

**Table:**

Species	Population	*n*	Voucher no.^a^	Collection locality	Geographic coordinates
*Oxytropis diversifolia* E. Peter	Baotoubei	20	Chang2016005	Baotou, Nei Mongol, China	40°42.953′N, 110°6.159′E
*O. diversifolia*	Pop8	30	Chang2016024^b^	Urad Zhongqi, Nei Mongol, China	41°24.063′N, 109°21.864′E
*O. diversifolia*	Hu	32	Chang2017034	Urad Zhongqi, Nei Mongol, China	41°34.163′N, 108°18.311′E
*O. diversifolia*	Dian1	32	Chang2017030	Urad Zhongqi, Nei Mongol, China	41°31.241′N, 107°37.741′E
*O. diversifolia*	Pop6	1	Chang2016022	Urad Zhongqi, Nei Mongol, China	41°29.463′N, 108°57.338′E
*O. diversifolia*	Damaonan	1	Chang2016015	Damao qi, Nei Mongol, China	41°25.867′N, 109°58.135′E
*O. diversifolia*	Chaogewenduoer	1	Chang2016076	Urad houqi, Nei Mongol, China	41°28.647′N, 106°57.054′E
*O. leptophylla* (Pall.) DC.	Bop2	19	Chang2016088	Guyang County, Nei Mongol, China	41°4.157′N, 110°6.252′E
*O. neimonggolica* C. W. Chang & Y. Z. Zhao	Yangcigoukou	20	Chang2017005	Alxa Zuoqi, Nei Mongol, China	39°1.897′N, 106°7.256′E
*O. squammulosa* DC.	Line	16	Chang2017043	Urad Zhongqi, Nei Mongol, China	41°24.063′N, 109°21.864′E

Note

*n* = number of individuals sampled; Chang = Zhao‐Yang Chang, group collection indicator.

a All voucher specimens are deposited in the Northwest A&F University Herbarium (WUK), Yangling, Shaanxi, China.

b Sample used for initial library construction.
